# High gain/bandwidth off-chip antenna loaded with metamaterial unit-cell impedance matching circuit for sub-terahertz near-field electronic systems

**DOI:** 10.1038/s41598-022-22828-3

**Published:** 2022-10-25

**Authors:** Mohammad Alibakhshikenari, Bal S. Virdee, Dion Mariyanayagam, Valeria Vadalà, Mohammad Naser-Moghadasi, Chan H. See, Iyad Dayoub, Sonia Aïssa, Patrizia Livreri, Shah Nawaz Burokur, Anna Pietrenko-Dabrowska, Francisco Falcone, Slawomir Koziel, Ernesto Limiti

**Affiliations:** 1 Department of Signal Theory and Communications, Universidad Carlos III de Madrid, 28911 Leganés, Madrid, Spain; 2 Center for Communications Technology, London Metropolitan University, London, N7 8DB UK; 3 Department of Physic, Milan-Bicocca University, Milan, Italy; 4 Department of Electrical and Computer Engineering, Science and Research Branch, Islamic Azad University, Tehran, 14778-93855 Iran; 5 School of Engineering and the Built Environment, Edinburgh Napier University, 10 Colinton Rd., Edinburgh, EH10 5DT UK; 6 Université Polytechnique Hauts-de-France, CNRS, University of Lille, ISEN, Institut d’électronique de Microélectronique et de Nanotechnologie, and INSA Des Hauts de France, Valenciennes, France; 7 Institut National de la Recherche Scientifique (INRS), University of Québec, Montréal, QC H5A 1K6 Canada; 8 Department of Engineering, University of Palermo, Viale delle Scienze BLDG 9, 90128 Palermo, Italy; 9 LEME, UPL, Univ Paris Nanterre, 92410 Ville d’Avray, France; 10 Faculty of Electronics, Telecommunications and Informatics, Gdansk University of Technology, 80-233 Gdansk, Poland; 11 Department of Electric, Electronic and Communication Engineering and the Institute of Smart Cities, Public University of Navarre, 31006 Pamplona, Spain; 12 School of Engineering and Sciences, Tecnologico de Monterrey, 64849 Monterrey, Mexico; 13 Engineering Optimization and Modeling Center, Reykjavik University, 101 Reykjavik, Iceland; 14 Electronic Engineering Department, University of Rome “Tor Vergata”, Via Del Politecnico 1, 00133 Rome, Italy

**Keywords:** Electrical and electronic engineering, Electronic and spintronic devices

## Abstract

An innovative off-chip antenna (OCA) is presented that exhibits high gain and efficiency performance at the terahertz (THz) band and has a wide operational bandwidth. The proposed OCA is implemented on stacked silicon layers and consists of an open circuit meandering line. It is shown that by loading the antenna with an array of subwavelength circular dielectric slots and terminating it with a metamaterial unit cell, its impedance bandwidth is enhanced by a factor of two and its gain on average by about 4 dB. Unlike conventional antennas, where the energy is dissipated in a resistive load, the technique proposed here significantly reduces losses. The antenna is excited from underneath the antenna by coupling RF energy from an open-circuited feedline through a slot in the ground-plane of the middle substrate layer. The feedline is shielded with another substrate layer which has a ground-plane on its opposite surface to mitigate the influence of the structure on which the antenna is mounted. The antenna has the dimensions 12.3 × 4.5 × 0.905 mm^3^ and operates across the 0.137–0.158 THz band corresponding to a fractional bandwidth of 14.23%. Over this frequency range the average measured gain and efficiency are 8.6 dBi and 77%, respectively. These characteristics makes the proposed antenna suitable for integration in sub-terahertz near-field electronic systems such as radio frequency identification (RFID) devices with high spatial resolution.

## Introduction

Terahertz (THz) technologies have lately attracted enormous interest in the research and commercial sectors for various applications such as noninvasive biomedical imaging^1^, detection of defects^2^, and short-range wireless communications^3^. The shorter THz wavelength, compared to that of microwave and millimeter waves, provides high spatial resolution, which is essential for biomedical imaging and defect detection. Moreover, the very large bandwidth available at the THz-band (0.1–1 THz) overcomes current issues of data rate and capacity limitations in wireless systems.

Existing THz technologies are bulky and expensive, which makes them unattractive for portable devices^4^. However, it is now possible to implement highly integrated THz systems on a chip using silicon-based CMOS process, which offers great advantages in terms of cost and ease of fabrication compared with compound semiconductors (e.g., III–V semiconductors). The critical component of any wireless system is the antenna because it interfaces the system to the propagating medium and is responsible for signal transmission and reception. The size of the antenna is related to the wavelength of operation. At THz band the shorter wavelength makes possible the development of miniature antennas suitable for integrating with the THz chip. However, implementation of the miniature antennas requires consideration of unwanted parasitic effects that can undermine the system performance, and the uncertainty introduced by wire or flip-chip bonding^5^, as the antenna is directly connected to the integrated circuit.

Maximum range of THz systems and how much power they can receive/capture depends on the gain of the antenna which in turn determines the equivalent isotropic radiated power (EIRP). The gain and radiation efficiency of on-chip antennas are severely affected by substrate loss, slots and thin dielectric layers^6^. In addition, surface waves that are induced on the antenna can distort the radiation pattern in terms of beamwidth as well as degrade its gain^7^. A simple technique in^8^ redirects the radiated signal back to the front-side by using a reflective surface under the silicon substrate on which the antenna is implemented. This technique is viable for broadside radiation antennas. However, the drawback of this technique is that it makes the antenna gain highly sensitive to the on-chip circuit vias and metal routings. To date, various other techniques have been explored to improve the performance of on-chip antennas, including the use of thinner substrates^7^, artificial magnetic conductors^9^, micromachining^10^, silicon lenses^11^, and dielectric resonators (DR)^12^. However, these techniques compromise the antenna gain performance and add complexity to their fabrication. In addition, DR antennas leak electromagnetic (EM) energy into the lossy CMOS silicon substrate, which degrades the antenna efficiency.

This paper presents a novel antenna design that exhibits a high gain and high efficiency over a wide operating bandwidth, while at the same time its miniature size makes it suitable for off-chip THz applications such as anti-counterfeiting security tags and quantum sensors^13^. The unique feature of the proposed antenna is a meandering transmission line structure which is loaded with multiple slots over its length and the line is terminated with metamaterial-inspired impedance matching circuit (MTM-IMC). The size and periodicity of the slots is subwavelength over the frequency band of operation. These characteristics transform the meandering line to a metasurface^14,15^. The proposed antenna is implemented on a multilayer stack of 400 μm thick silicon substrate (*ε*_r_ = 11.9). The antenna is excited with an open-circuited microstrip line by coupling RF energy through a slot, which is implemented on a ground-plane sandwiched between two silicon layers. Such a THz antenna can provide high spatial resolution that overcomes the limitation of for example RFID tags in their ability to resolve other RFID devices in close proximity^16^.

## Off-chip antenna design

Metamaterials (MTM) exhibit a negative refractive index, and a metamaterial unit cell can be modelled using an equivalent electrical T-circuit comprising series capacitors and a shunt inductor^17^. The negative refractive index exhibited by the metamaterial is due to the resonance of the unit cell, which makes the metamaterials inherently dispersive. The electromagnetic properties of such materials are highly sensitive to the changes in the operating frequency, which makes them bandwidth limited. Metasurfaces are two-dimensional or surface counterparts of metamaterials. Metasurfaces can be implemented using an array of subwavelength resonant scatters consisting of dielectric slots constructed on a transmission line^14,15^. The size and periodicity of the individual slots need to be small in comparison to the wavelength of operation, which is different from frequency selective surfaces (FSS) where individual elements are spaced by λ/2 at the operating wavelength. It has been shown in^18^ that the dispersion characteristics of metasurfaces are broadened with multiple scatterers. This principle is applied in the proposed antenna design.

**Figure 1:**
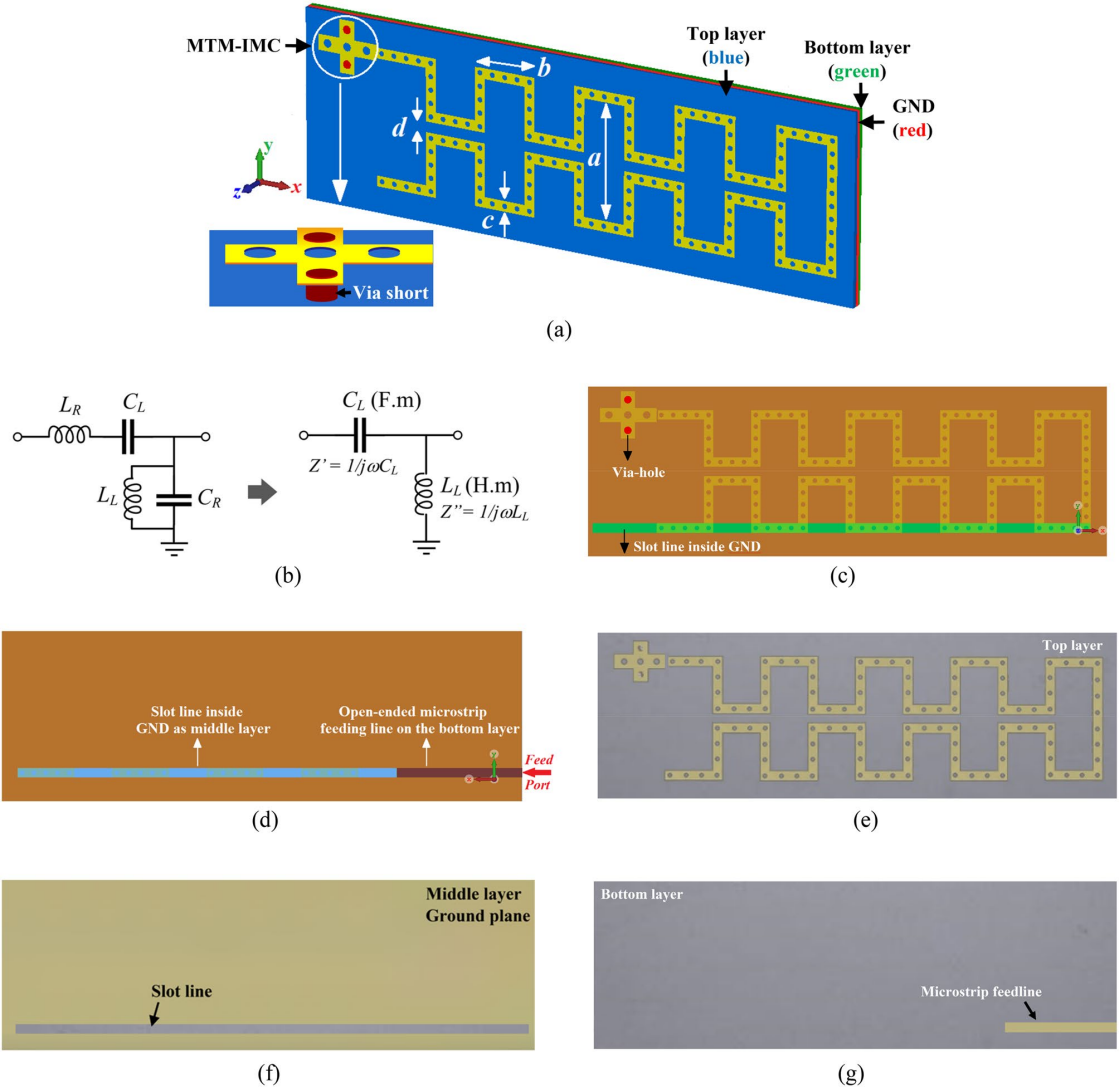
Proposed on-chip antenna, (**a**) simulated isometric view of the OCA structure showing a close-up view of the MTM-IMC, (**b**) equivalent circuit model of the MTM-IMC, (**c**) top-view of the antenna layout along with an overview of the coupling slot line on the ground plane showing its precise location underneath the slotted meandering line, (**d**) back-side of the simulated layout with an overview of the coupling slot line on the ground plane showing its precise position in relation to the open-ended microstrip feedline, (**e**) top-view of the fabricated prototype OCA, (**f**) fabricated middle ground-plane layer (GND) with coupling slot line, and (**g**) fabricated back-side of the OCA showing the open-ended microstrip feedline, which is shielded with a substrate layer whose outward face side is a ground-plane.

The proposed off-chip antenna (OCA) is constructed on a stack of three silicon layers. The antenna is realized on the surface of the top layer. The bottom side of the top silicon layer is a ground-plane on which a narrow slot line is created. On the bottom side of the second silicon layer is a feed structure consisting of an open-circuited microstrip line which is aligned with the slot line and the input port to the antenna. Stacked over the feedline is a substrate layer whose outward facing side is ground-plane to shield the OCA from being susceptible to materials on which it is mounted on. The radiating element of the OCA is an elongated and folded open-circuited loop transmission line whose other end is terminated with a metamaterial-inspired impedance matching circuit, as shown in Fig. 1. Although by meandering the antenna some discontinuity is created however this was necessary to reduce its footprint. The meandering antenna is loaded with a series of regularly spaced circular dielectric slots along its length. The size and periodicity of the individual slots is smaller than the wavelength of operation. The slots act like subwavelength resonant scatters that transforming the meandered line into a metasurface^14,15^. The antenna is excited from underneath by coupling RF energy from the open-circuited feedline through the rectangular slot implemented on the middle ground-plane layer.

Unlike standing wave antennas, the circular slots implemented on the meandering line antenna do not need to be excited in-phase. Consequently, the gap between the slots do not depend on the wavelength of the signal. It will be shown later that this results in a structure that exhibits a higher impedance bandwidth compared to the standing wave structure.

The MTM-IMC is a cross-shaped microstrip structure with slots, as shown in Fig. 1. It is capacitively coupled to the antenna and is grounded with metallized via-holes. This structure is a left-handed metamaterial whose equivalent electrical circuit consists of a series left-handed capacitance (*C*_*L*_) and shunt left-handed inductance (*L*_*L*_)^17^. The MTM-IMC structure has unavoidable fringing fields resulting from the currents flowing on the surface of the antenna and the gap between the surface and the ground-plane. Hence, the fringing fields create right-handed parasitic elements of series right-handed inductances (*L*_*R*_) and shunt right-handed capacitances (*C*_*R*_) associated with the left-handed components. The equivalent circuit of the MTM-IMC is shown in Fig. 1b. The magnitude of the reactive components resulting from the fringing fields are negligible and can be therefore ignored in the analysis. The equivalent circuit then simplifies to series *C*_*L*_ and shunt *L*_*L*_. For simplicity, assuming loss-less case, the propagation constant *γ*, the propagation constant *β*, the phase velocity *v*_*p*_, and the group velocity *v*_*g*_ of the transmission-line can be shown to be given by

1 $$\gamma = j\beta = \sqrt {Z^{\prime } Y^{\prime \prime } } = - j1/\omega \sqrt {L_{L} C_{L} }$$

2 $$\beta = - 1/\omega \sqrt {L_{L} C_{L} }$$

3 $$v_{p} = \omega /\beta = - \omega^{2} \sqrt {L_{L} C_{L} } < 0$$

4 $$v_{g} = \left( {\partial \beta /\partial \omega } \right)^{ - 1} = + \omega^{2} \sqrt {L_{L} C_{L} } > 0$$

Eqs. (3) and (4) show that the phase velocity, associated with the direction of phase propagation, is negative, whereas the group velocity, associated with the direction of power flow, is positive. These characteristics are manifestation of metamaterials. The geometrical parameters of the proposed OCA are listed in Table 1. Its overall physical dimension is 12.3 $\times \hspace{0.17em}$4.5 $\times \hspace{0.17em}$0.905 mm^3^.

**Table 1:** Structural dimensions of OCA ($\mathrm{unit}: \mathrm{\mu m}$).

Thickness of silicon substrate	400
Thickness of metalized ground-plane layer	35
Thickness of microstrip conductor	35
Radius of circular MTM-IMC slots	85
Gap between the MTM-IMC slots	340
Radius of meander line slots	45
Gap between the meander line slots	300
Radius of metallic via-holes	85
Gap between the via-holes and slots	330
Length of the ground-plane slot	11,900
Width of the ground-plane slot	380
Length of the microstrip feedline	3500
Width of the microstrip feedline	380
Parameter *a* in Fig. 1a	2677
Parameter *b* in Fig. 1a	1151
Parameter *c* in Fig. 1a	380
Parameter *d* in Fig. 1a	375

## Measured results

**Figure 2:**
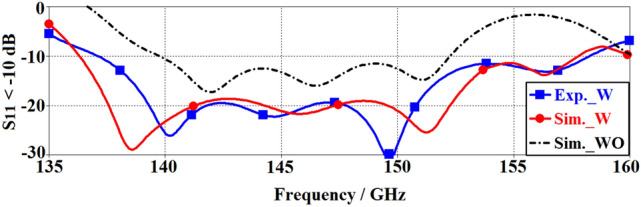
Simulated and measured reflection-coefficient response of the proposed OCA without (WO) and with (W) the MTM-IMC termination and slots.

The simulated and measured reflection-coefficient response of the proposed OCA are compared in Fig. 2 with and without the MTM-IMC and slots. The simulation was done with the 3D full-wave EM tool CST Microwave Studio.

The simulation results in Fig. 2 show that without MTM-IMC plus slots, the antenna’s impedance bandwidth for S_11_ < − 10 dB is 0.012 THz (0.14–0.152 THz), which corresponds to a fractional bandwidth of 8.21%. With MTM-IMC plus slots the measured impedance bandwidth is significantly improved by a factor of approximately 2–0.021 THz (0.137–0.158 THz), which corresponds to a fractional bandwidth of 14.23%. Moreover, across 0.14–0.152 THz range, the average impedance matching with MTM-IMC plus slots is better than -21 dB, whereas without MTM-IMC plus slots it is limited to − 13 dB. The empirical results clearly demonstrate a significant improvement in the reflection coefficient by 8 dB, and this is an indication of its effectiveness in suppressing losses attributed to surface waves and substrate losses. Figure 2 also shows very good agreement between the simulated and measured results.

**Figure 3:**
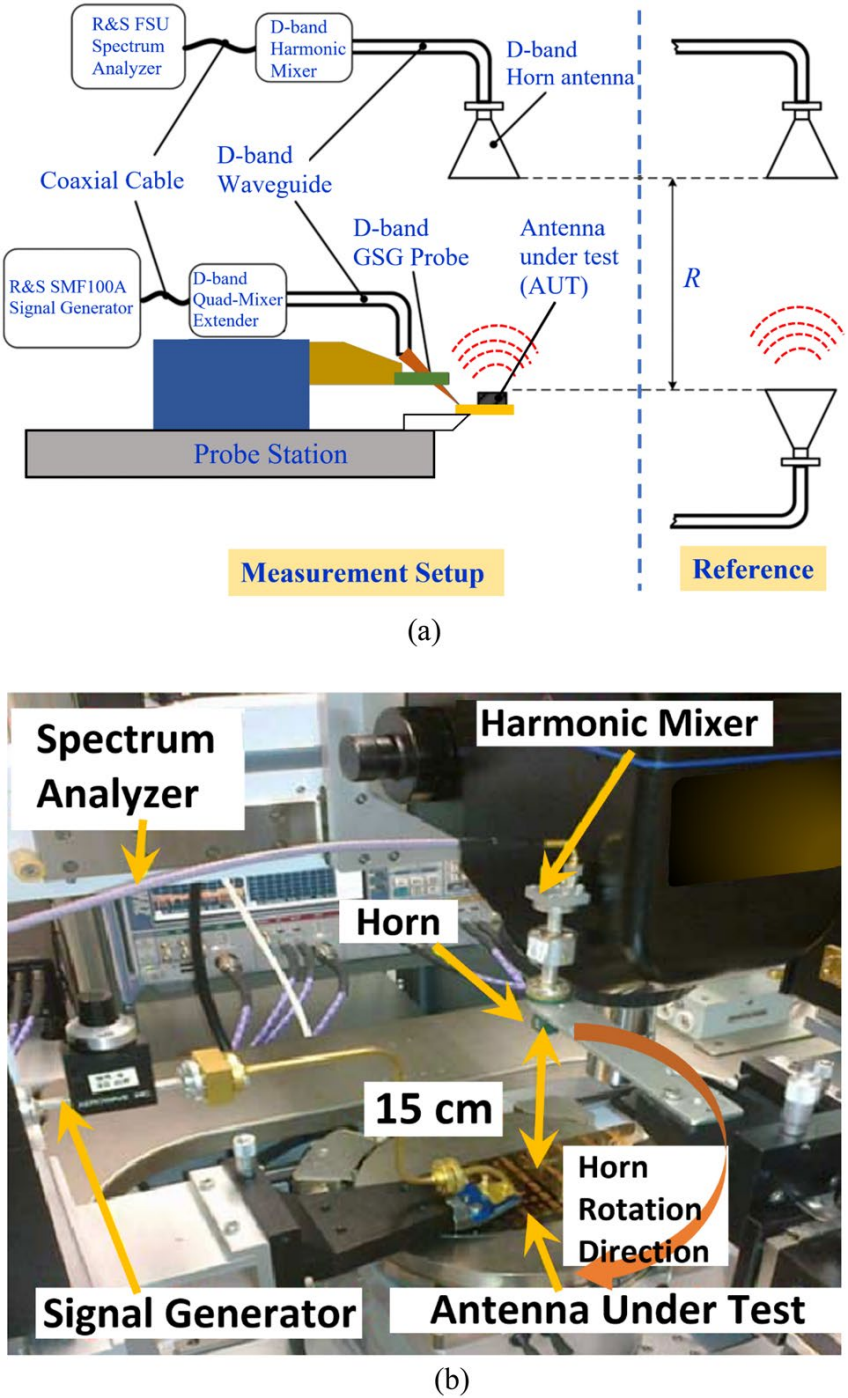
(**a**) Diagram of the on-wafer measurement setup for the power gain test. (**b**) Photograph of the on-wafer gain measurement setup.

In addition to the impedance bandwidth, the other characteristics that define the antenna’s performance are the gain and radiation efficiency. The measurement of these parameters for the OCA required a probe station setup as described in^19^, which is illustrated in Fig. 3a. The photograph of the setup used is shown in Fig. 3b. The signal from the R&S SMF 100A was applied to a D-band quad-mixer extender. The transmission signal from the mixer is fed to the OCA using a D-band waveguide-to-GSG probe. The received signal at the D-band standard horn antenna is down-converted through a harmonic mixer and fed to the R&S SMF Spectrum Analyzer for measurement. The horn antenna was used to sense the radiation from the OCA at a far-field distance of 15 cm. The OCA was then replaced by another standard horn antenna, and the antenna gain was obtained using the traditional method of comparing the power received by the standard horn of a known gain with that received by the OCA. Measurements done consider the waveguide-to-GSG probe loss of ~ 1.6 dB and the waveguide-to-horn transition loss of ~ 1 dB.

**Figure 4:**
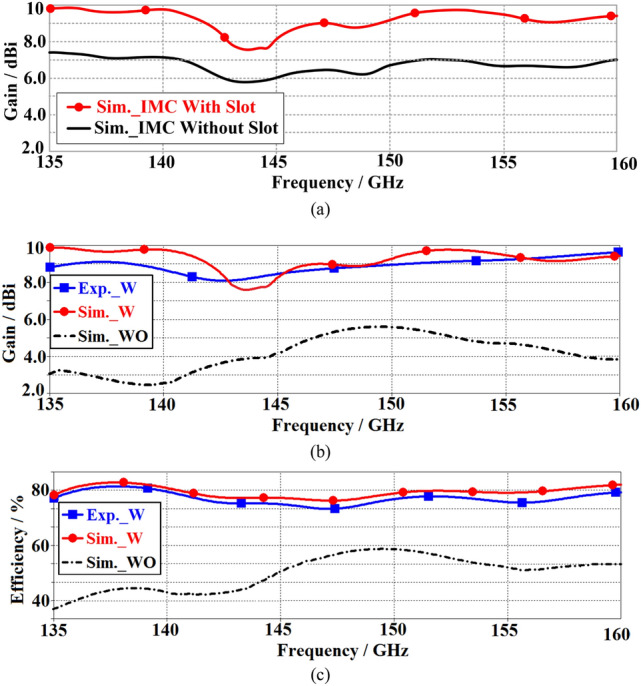
(**a**) Simulated gain of the OCA under two conditions, (1) MTM-IMC and without (WO) slot, and (2) MTM-IMC and with (W) slots, (**b**) simulated and measured gain of the OCA before and after applying MTM-IMC and slots, and (**c**) simulated and measured efficiency of the OCA before and after applying MTM-IMC and slots radiation efficiency.

The directivity/gain method was used to measure the radiation efficiency using the expression *η* = *G*/*D*, where *G* is the gain of the OCA measured using a standard gain horn, and *D* is the directivity of OCA. Directivity was determined from the expression *D* = 41,253/(ΔθΔϕ), where Δθ and Δϕ are the principal plane beamwidths (in decimal degrees) measured from the radiation pattern of the antenna^20^. The equipment other than the OCA and horn antenna was covered with radiation absorbent material (RAM) to prevent the reflections from affecting the measurements. The OCA’s back lobe was measured by rotating the horn antenna under the OCA, but this was limited due to the measurement setup used. Figure 4a shows the simulated gain of the OCA including MTM-IMC under two scenarios, with slots and without slots. It is evident that with the slots there is a gain improvement of about 3 dB. The measured gain and radiation efficiency of the OCA before and after applying MTM-IMC and slots are shown in Fig. 4b,c, respectively. These results show good agreement between the simulation and measurement. The average gain and efficiency prior to implementing the proposed MTM-IMC plus slot is 4 dBi and 46%, respectively. After applying the MTM-IMC plus slot, the measured gain and efficiency significantly increase to 8.6 dBi and 77%, respectively. This constitutes an improvement of 4.6 dBi and 31% in the gain and the efficiency, respectively. These results are summarized in Table 2.

**Table 2:** Radiation characteristics.

	Gain (dBi)	Efficiency (%)
**Without MTM-IMC plus slots (simulated)**		
Maximum	5.6 @ 149 GHz	59 @ 150 GHz
Minimum	2.5 @ 140 GHz	37.5 @ 135 GHz
Average	4	46
**With MTM-IMC plus slots (measured)**		
Maximum	9.6 @ 158 GHz	81 @ 137.5 GHz
Minimum	8.1 @ 143 GHz	74 @ 147.5 GHz
Average	8.6	77
**Improvement with MTM-IMC plus slots**		
Average	4.6	31

**Figure 5:**
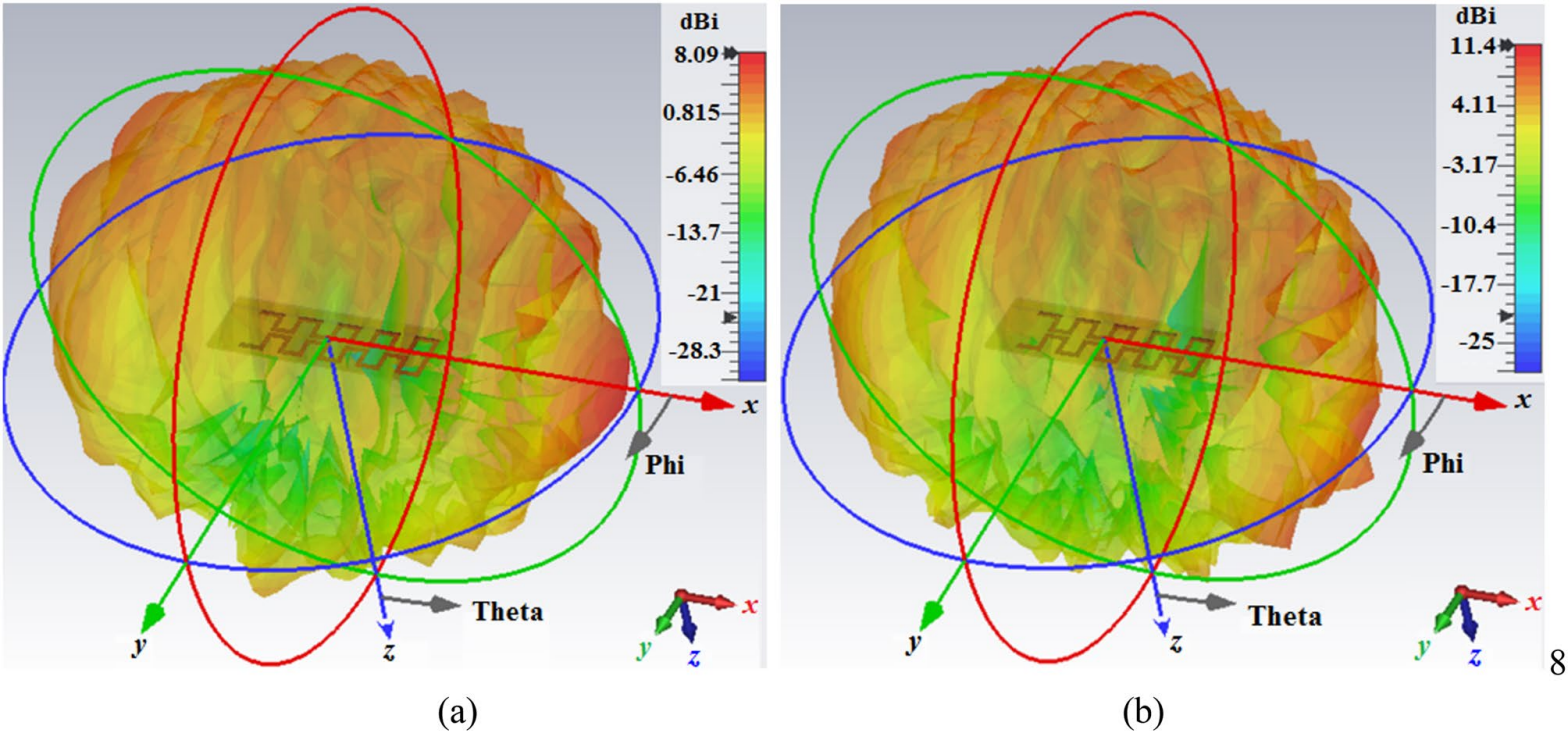
Radiation patterns of the proposed OCA with the MTM-IMC termination and slots at (**a**) 0.137 THz, and (**b**) 0.158 THz.

**Figure 6:**
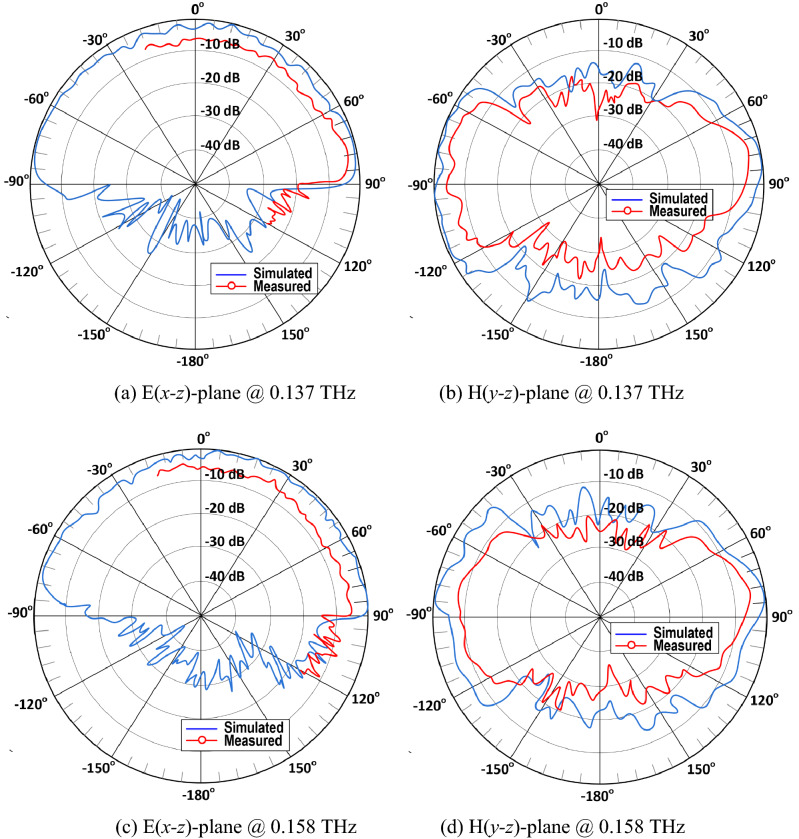
Normalized simulated and measured radiation patterns in the E-plane and H-plane of the proposed OCA with the MTM-IMC termination and slots.

The simulated radiation patterns of the proposed OCA with the MTM-IMC termination at two spot frequencies in its operating band are shown in Fig. 5. This antenna radiates in the broadside over its entire length. The simulated and measured radiation patterns in the two orthogonal planes are shown in Fig. 6. The radiation coverage in the E(*x–z*)-plane is 180° along the length of the antenna. However, the H(*y–z*)-plane radiation pattern is attenuated at its sides. These results show that changes in frequency does not cause significant changes in the radiation patterns. Also, there is good correlation between the simulated and measured results.

**Figure 7:**
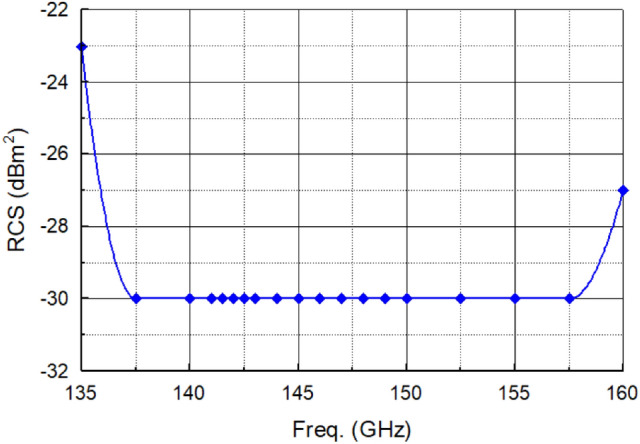
Measured radar cross section (RCS) of the proposed OCA tag.

The radar cross section (RCS) of OCA that characterizes its backscattering was measured. The measured RCS for the proposed OCA, in Fig. 7, was calculated using the expression $RCS={|{S}_{11}|}^{2}\left[{\left(4\pi \right)}^{3}{r}^{4}/{\left(\lambda G\right)}^{2}\right]$, where S_11_ is the magnitude of the reflection coefficient at wavelength λ, *G* is the gain of the transmitting antenna, and *r* is the distance to the tag antenna^21,22^. RCS is better than − 23 dBm^2^ from 135 to 160 GHz.

## Comparison with prior research

In this section, the performance of the proposed OCA is compared with other antennas that can be integrated in sub-THz and THz wireless systems. The comparison shown in Table 3 confirms that the proposed antenna structure having a fractional bandwidth of 14.23% supports a wider operational bandwidth than other antennas except for references^23–26^. Moreover, the proposed OCA has the highest average gain and efficiency performances. Although the dimensions of the proposed antenna are bigger than other cited works, its structure is less complex to implement in practice, and therefore it is more cost effective. These characteristics make the antenna viable for sub-THz integrated RFID circuits for wireless applications. The present work was a feasibility study. However, in the future we intend to reduce the antenna size by decreasing the gap between the folds of the meandering line, and by using a substrate with a higher permittivity.

**Table 3:** Performance comparison.

References	Type	FBW (%)/[Freq. range (GHz)]	Ave. Gain (dBi)	Max. Gain (dBi)	Eff. (%)	Size (mm^3^)	Complexity
^23^	Bowtie-slot	15.38/[90–105]	− 2.2	− 1.78 @ 98 GHz	–	0.71 × 0.31 × 0.65	High
^24^	Circular open-loop	16.13/[57–67]	− 5.0	− 4.4 @ 66 GHz	–	1.8 × 1.8 × 0.3	High
^27^	Dipole-antenna	7.1/[95–102]	–	4.0 @ 94 GHz	–	–	Moderate
^28^	Cavity fed DRA	11.32/[125–140]	4.0	7.5 @ 133 GHz	Max. 42	0.8 × 0.9 × 2.2	High
^19^	Slot fed DRA	7.7/[125–135]	2.0	4.7 @ 125 GHz	Max. 43	0.9 × 0.8 × 1.5	High
^25^	DRA	15.38/[120–140]	− 2.0	4.1 @ 125 GHz	Max. 43	0.9 × 0.8 × 0.6	High
^29^	4 × 1 Patch array	11.63/[259–291]	–	11.2 @ 275 GHz	–	2.47 × 1.53 × 0.67	Moderate
^30^	2 × 1 Shorted annular ring	5.45/[303–320]	1.0	5.0 @ 315 GHz	Max. 38	0.55 × 1 × 0.13	High
^26^	Dipole loaded AMC	33.67/[200–281]	0	2.0 @ 96 GHz	Max. 63	0.25 × 0.41 × 0.13	Moderate
^31^	2 × 2 slot array	2.74/[323–332]	3.0	7.9 @ 320 GHz	–	0.86 × 0.86 × 0.13	High
^32^	2 × 2 DRA array	2.34/[334–350]	1.0	8.65 @ 338 GHz	54	2.2 × 1.4 × 0.65	High
This work	OCA with MTM-IMC	14.23/[137–158]	8.6	9.6 @ 158 GHz	Max. 81 @ 137.5 GHz	12.3 × 4.5 × 0.905	Low

## Conclusion

The proposed meandering line THz antenna structure is shown to exhibit high gain and high efficiency performance. The antenna structure was fabricated on a stack of three silicon layers and consists of a single meandering line that is loaded with a series of regularly spaced circular slots along its length and terminated by a metamaterial-based impedance matching circuit. The antenna radiates power through the circular slots when it is excited using an open-ended microstrip line realized on the bottom silicon layer. Electromagnetic energy is coupled from the feedline to the top silicon layer through a slot line etched in the middle ground-plane layer. The measurements confirm that the fabricated antenna has a relatively large fractional bandwidth of 14.23%, a peak gain of 9.6 dBi and the maximum radiation efficiency of 81%. Such characteristics of wide bandwidth, high gain, and high efficiency makes the antenna an excellent candidate for integrating with sub-terahertz near-field electronic systems.

## Data Availability

All data generated or analysed during this study are included in this article.
